# Supplementary material for: Suppression of TGF-β/SMAD signaling by an inner nuclear membrane phosphatase complex
Source: Nat Commun. 2025 Apr 11;16:3474. doi: 10.1038/s41467-025-58681-x (PMC11992160; doi:10.1038/s41467-025-58681-x)
Supplement: Supplementary file 1 — Supplementary Information [file 41467_2025_58681_MOESM1_ESM.pdf]

Supplementary Information

Suppression of TGF-β/SMAD signaling by an inner nuclear membrane phosphatase complex

Zhe Ji[1,*], Wing-Yan Skyla Siu[1,*], Maria Emilia Dueñas[2,3], Leonie Müller[2], Matthias Trost[2], Pedro Carvalho[1,§]

[1]Sir William Dunn School of Pathology, University of Oxford, South Parks Road, Oxford, OX1 3RE, UK

[2]Biosciences Institute, Newcastle University, Framlington Place, Newcastle upon Tyne, NE2 4HH, UK

[3]Current address: Telethon Kids Institute, Perth, Nedlands WA 6009, Australia

[*]These authors contributed equally to this work

[§]Lead Contact

Correspondence should be sent to:

Pedro Carvalho (pedro.carvalho@path.ox.ac.uk; phone: +44 1865 618 654)

This PDF file includes

Supplementary Fig. 1-5

Supplementary Table 1

Supplemental Figure 1. Localization and immunoprecipitation analysis of CTDNEP1, NEP1R1 and MAN1

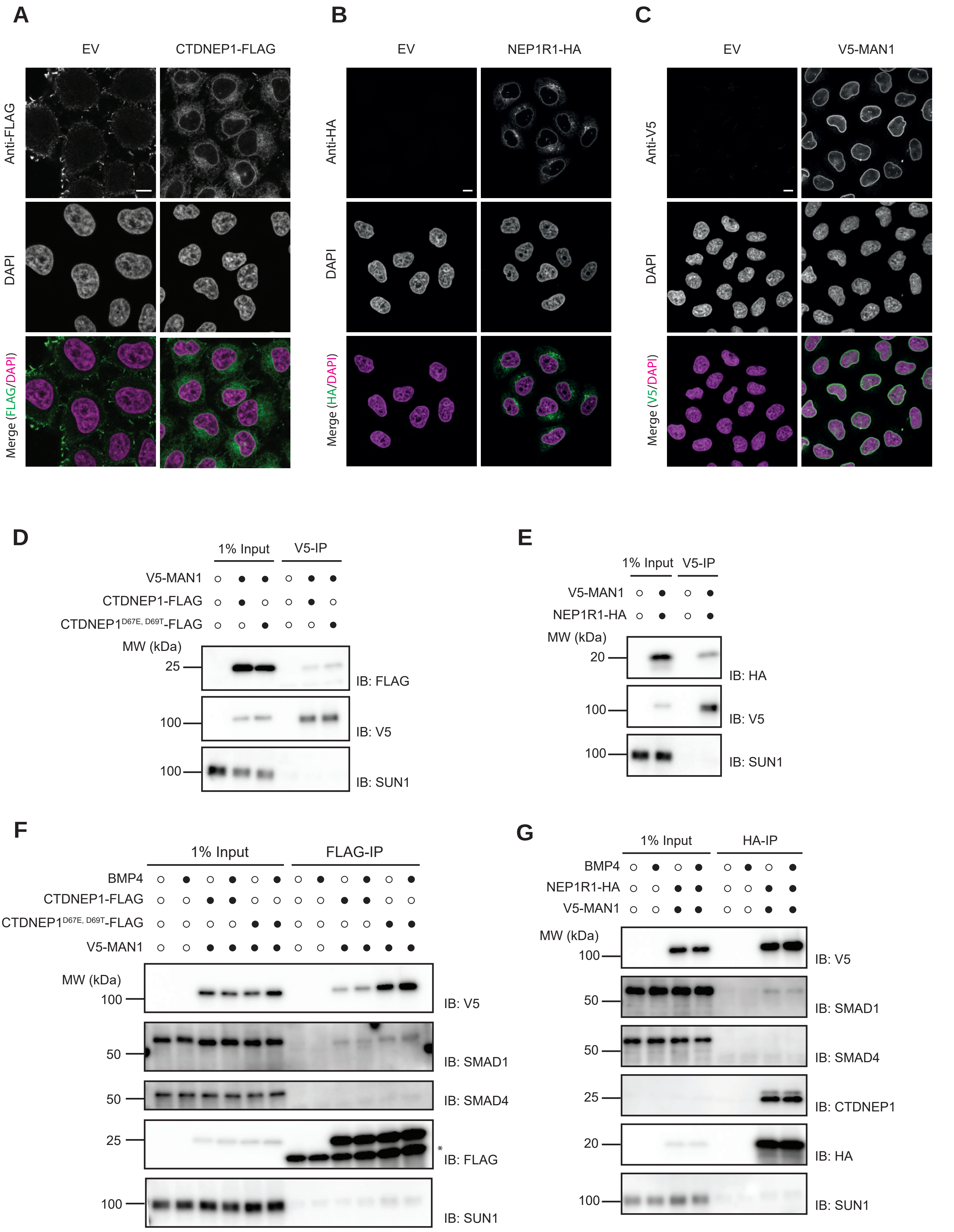

Figure S1. Localization and immunoprecipitation analysis of CTDNEP1, NEP1R1 and MAN1

Localization of CTDNEP1-FLAG (A), NEP1R1-HA (B) and V5-MAN1 (C) in HeLa cells analyzed by immunofluorescence. CTDNEP1, NEP1R1 and MAN1 were detected with anti-FLAG, -HA and –V5 antibodies, respectively and DNA was labelled with 4',6- diamidino- 2- phenylindole (DAPI). Cell transduced with an empty vector (EV) were used as control. Note that anti-FLAG antibody non-specifically labels the cell periphery. Scale bar:10μM.

(D and E) Immunoprecipitation of V5-MAN from detergent solubilized extracts of HeLa cells co-expressing either CTDNEP1 or CTDNEP1$^{D67E, D69T}$-FLAG (D) or NEP1R1-HA (E). Eluted proteins were analyzed by SDS-PAGE followed by immunoblotting with the indicated antibodies.

(F and G) Immunoprecipitation of CTDNEP1 or CTDNEP1$^{D67E, D69T}$-FLAG (F) or NEP1R1-HA (G) from detergent solubilized extracts of HeLa cells co-expressing V5-MAN1. Immunoprecipitations were performed in absence or upon 1hr treatment with 20ng/ml of BMP4.

Eluted proteins were analyzed by SDS-PAGE followed by immunoblotting with the indicated antibodies. The asterisk (*) indicates the light chain of the antibody used for immunoprecipitation

Supplemental Figure 2.R-SMAD dephosphorylation requires MAN1 and the CTDNEP1-NEP1R1 phosphatase in U2OS cells

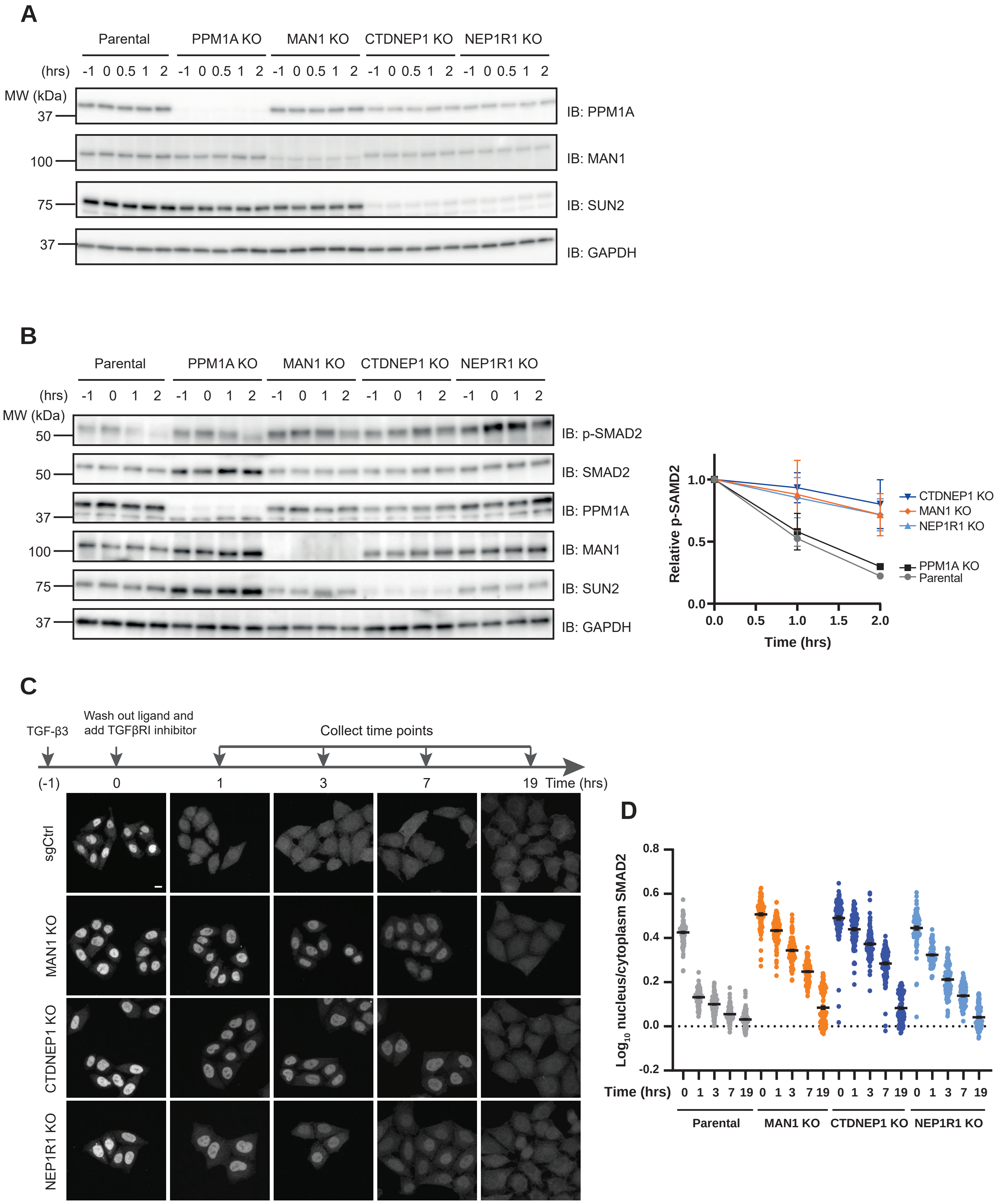

Figure S2. R-SMAD dephosphorylation requires MAN1 and the CTDNEP1-NEP1R1 phosphatase in U2OS cells

(A) Validation of PPM1A, MAN1, CTDNEP1 and NEP1R1 knock out HeLa cells. Cell extracts were analyzed by SDS-PAGE followed by immunoblotting with the indicated antibodies.

(B) Time course analysis of SMAD2 dephosphorylation upon TGF-β3 stimulation in parental, PPM1A, MAN1, CTDNEP1 and NEP1R1 KO U2OS cells. Cell lysates were subjected to SDS-PAGE separation and immunoblotting was performed with the indicated antibodies. The graph (right) shows the average of three experiments; error bars represent standard deviation.

(C) Immunofluorescence of time course analysis of endogenous SMAD2 localization upon TGF-β3 stimulation in parental, MAN1 KO, CTDNEP1 KO and NEP1R1 KO HeLa cells.

(D) Quantification of nuclear accumulation of SMAD2 from imaging experiments as shown in (C) from three independent biological replicates. Error bars represent the standard error mean of the three replicates ($n > 90$).

Supplemental Figure 3.R-SMAD dephosphorylation requires CTDNEP1-NEP1R1 phosphatase

A

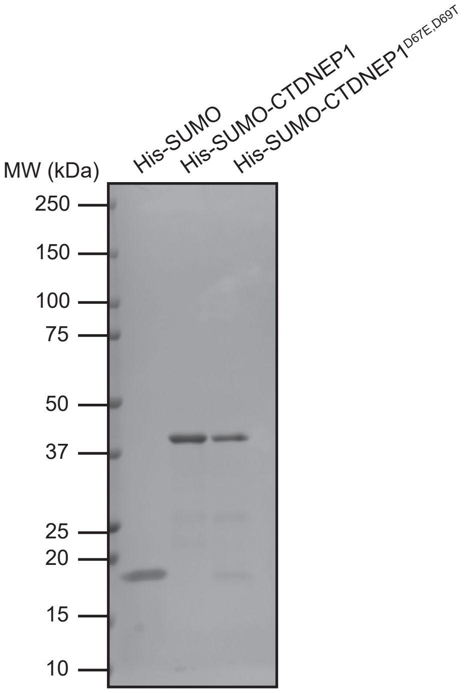

B

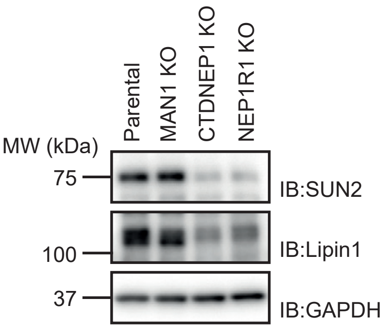

Figure S3. R-SMAD dephosphorylation requires CTDNEP1-NEP1R1 phosphatase

(A) Purified His-SUMO tagged wild type or phosphatase dead CTDNEP1, or His-SUMO alone. The purity of the recombinantly expressed proteins was analyzed by SDS-PAGE followed by staining with Coomassie blue. Note that soluble versions of CTDNEP1 and of CTDNEP1^{D67E,D69T} were generated by deleting the N terminal amphipathic helix of CTDNEP1 corresponding to amino acids 1-45.

(B) Steady state levels of Lipin and SUN2 are specifically affected by the loss of CTDNEP1 and NEP1R1 while loss of MAN1 has no effect. Extracts of HeLa cells with the indicated genotype were analyzed by SDS-PAGE followed by immunoblotting with anti-Lipin1 and anti-SUN2 antibodies. GAPDH was used as a loading control and detected with an anti-GAPDH antibody.

Supplemental Figure 4. Different MAN1 domains interact with NEP1R1 and R-SMADs

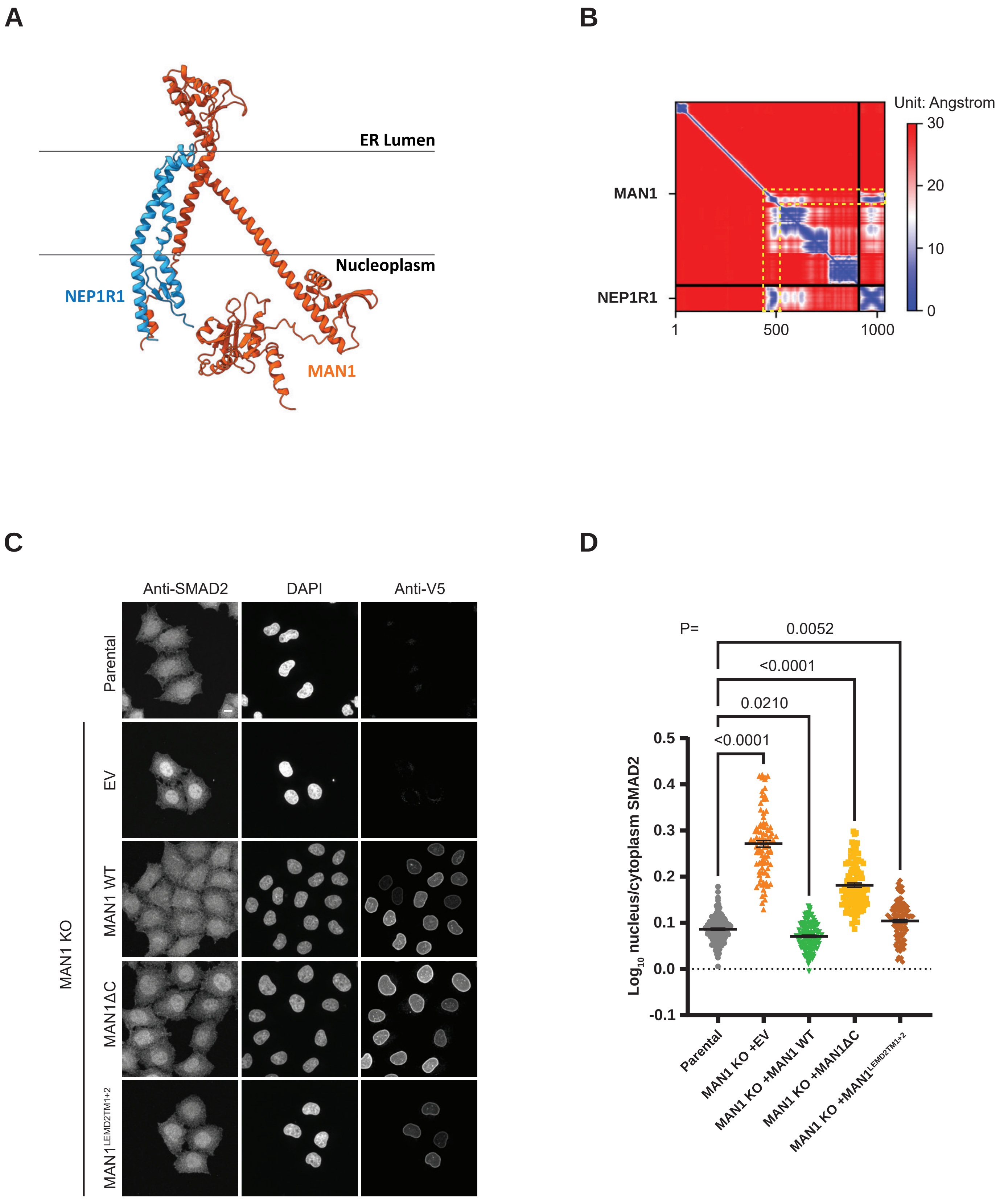

Figure S4. Different MAN1 domains interact with NEP1R1 and R-SMADs

(A) AlphaFold multimer structural model of NEP1R1 (light blue) and MAN1 (orange).

(B) Alphafold predicted alignment error (PAE) plot of the model shown in (A). The predicted interaction between MAN1 and NEP1R1 membrane regions is indicated by the dotted yellow box.

(C) Localization of endogenous SMAD2 in HeLa parental and MAN1 KO cells expressing the indicated V5-tagged MAN1 derivatives analyzed immunofluorescence. Cells transduced with an empty vector (EV) were used as control. SMAD2 and MAN1 derivatives were detected with anti-SMAD2 and anti-V5 antibodies, respectively. DNA was labelled with 4',6- diamidino- 2-phenylindole (DAPI). Scale bar:10µM

(D) Quantification of nuclear accumulation of SMAD2 from imaging experiments as shown in (C). n = 3 independent experiments, p values were indicated in the graph. One way ANOVA (multiple comparison) was performed and data are presented as mean values +/- SD.

Supplemental Figure 5. CTDNEP1, NEP1R1 suppress aberrant SMAD signaling

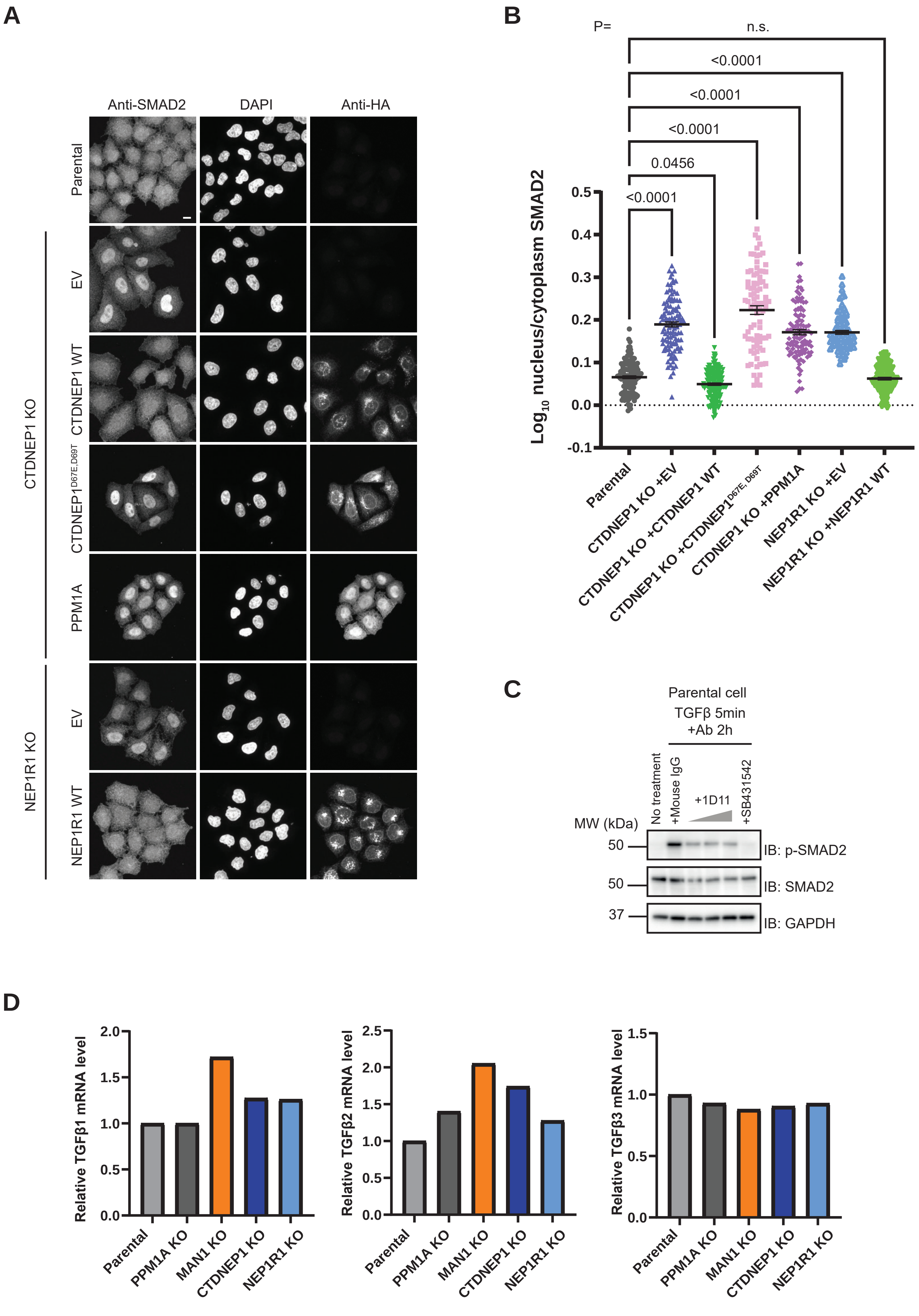

Figure S5. CTDNEP1, NEP1R1 suppress aberrant SMAD signaling

(A) Localization of endogenous SMAD2 in HeLa parental, CTDNEP1 KO or NEP1R1 KO cells expressing the indicated HA-tagged proteins analyzed immunofluorescence. Cells transduced with an empty vector (EV) were used as control. SMAD2 was detected with an anti-SMAD2 antibody and CTDNEP1 derivatives and NEP1R1 were detected with an anti-HA antibody. DNA was labelled with 4',6- diamidino- 2- phenylindole (DAPI). Scale bar:10μM

(B) Quantification of nuclear accumulation of SMAD2 from imaging experiments as shown in (A). n = 3 independent experiments, p values were indicated in the graph. One way ANOVA (multiple comparison) was performed and data are presented as mean values +/- SD.

(C) Levels of endogenous p-SMAD2 and SMAD2 in HeLa parental cells with indicated treatments. Mouse IgG antibody was used at 300 μg/mL, 1D11 antibody was used at 30, 150 and 300 μg/mL respectively. Cell lysates were analyzed by SDS-PAGE followed immunoblotting with the indicated antibodies. GAPDH was used as loading control.

(D) Levels of TGF-β1, 2 and 3 transcripts in HeLa parental cells or lacking the indicated genes analyzed by RT-qPCR.

Supplementary Table 1 - List of reagents used in this study

REAGENT or RESOURCE	SOURCE	IDENTIFIER
**sgRNA sequences**		
PPM1A: ATCGCCAGAAGCAGTGAAGA	This study	
LEMD3: ACAGCAACTCTGCAGAGCGA	This study	
CTDNEP1: GAGATCTATGGCTCTGCTG	This study	
NEP1R1: GCCGCCCGACATGAACTCGCAGG	This study	
**Recombinant DNA**		
Lentiviral cDNA expression vectors	Van de Weijer et al 2014	
Lentiviral CRISPR/Cas9 vector	Van de Weijer et al 2014	
pMD2.G	Addgene	12259
psPAX2	Addgene	12260
HIS14-sumo-CTDNEP1 WT (46-244)	This study	
HIS14-sumo-CTDNEP1 D67E, D69T(46-244)	This study	
**RT-qPCR primers**		
p21 For: AGGTGGACCTGGAGACTCTCAG	This study	
p21 Rev: TCCTCTTGGAGAAGATCAGCCG	This study	
p15 For: ACGGAGTCAACCGTTTCGGGAG	This study	
p15 Rev: GGTCGGGTGAGAGTGGCAGG	This study	
TGFβ1 For: GCCCTGGACACCAACTATTG	This study	
TGFβ1 Rev: CGTGTCCAGGCTCCAAATG	This study	
TGFβ2 For: AAGCTTACACTGTCCCTGCTGC	This study	
TGFβ2 Rev: TGTGGAGGTGCCATCAATACCT	This study	
TGFβ3 For: TCAGCCTCTCTCTGTCCACTT	This study	
TGFβ3 Rev: CATCACCGTTGGCTCAGGG	This study	
**Antibodies**		
Rabbit Monoclonal anti-SMAD2	Cell signaling	5339S RRID:AB_10626777
Rabbit Monoclonal anti-pSMAD2	Cell signaling	3108S RRID:AB_490941

Rabbit Monoclonal anti-SMAD1	Cell signaling	6944S<br>RRID:AB_10858882
Rabbit Monoclonal anti-pSMAD1/5/9	Cell signaling	13820S<br>RRID:AB_2493181
Mouse Monoclonal anti-FLAG-HRP (M2)	Merck Life Science UK Limited	A8592<br>RRID:AB_439702
Rat Monoclonal anti-HA (3F10)	Roche	11867423001<br>RRID:AB_390918
Mouse Monoclonal anti-GAPDH (1E6D9)	ProteinTech	60004-1-Ig<br>RRID:AB_2107436
Rabbit Monoclonal anti-V5	Cell signaling	13202S<br>RRID:AB_2687461
Rabbit Monoclonal anti-SMAD4	Cell signaling	38454S<br>RRID:AB_2728776
Rabbit Monoclonal anti-SUN1	Abcam	ab124770<br>RRID:AB_10976056
Rabbit Polyclonal anti-SUN2	Atlas Antibodies	HPA001209<br>RRID:AB_1080465
Rabbit Polyclonal anti-MAN1	Atlas Antibodies	HPA076986<br>RRID:AB_2686821
Rabbit Monoclonal anti-p21	Cell signaling	2947S<br>RRID:AB_823586
Rabbit Monoclonal anti-Lipin1	Cell signaling	5195S<br>RRID:AB_10694491
Peroxidase AffiniPure Goat Anti-Mouse IgG, light chain specific	Jackson ImmunoResearch	115-035-174,<br>RRID:AB_2338512
Peroxidase IgG Fraction Monoclonal Mouse Anti-Rabbit IgG, light chain specific	Jackson ImmunoResearch	211-032-171<br>RRID:AB_2339149
Peroxidase AffiniPure Goat Anti-Rat IgG, light chain specific	Jackson ImmunoResearch	112-035-175<br>RRID:AB_2338140
Self-raised anti-CTDNEP1 antibody	Eurogentec	
**Chemicals, Peptides, and Recombinant Proteins**		
DMEM medium	Merck Life Science UK Limited	D6429
Fetal Calf Serum	Merck Life Science UK Limited	F9665
L-Glutamine (200 mM)	Gibco (Thermo Fisher Scientific)	25030024
Penicillin-Streptomycin (10,000 U/mL)	Gibco (Thermo Fisher Scientific)	15140122

Zeocin	Invitrogen (Thermo Fisher Scientific)	R25001
Puromycin	Gibco (Thermo Fisher Scientific)	A1113803
Doxycycline	Merck Life Science UK Limited	D9891
TransIT LT1	Mirus Bio LLC	MIR 2305
OptiMEM	Gibco (Thermo Fisher Scientific)	31985062
Trypsin	Thermo Fisher Scientific	15090046
cOmplete EDTA-free protease inhibitor cocktail	Roche	5056489001
Benzonase	Merck Life Science UK Limited	E1014
1,4-Dithiothreitol (DTT)	Merck Life Science UK Limited	D9779
TGF-β3	Cell signaling	8425
TGF-β3	Cell signaling	10858
BMP-4	Peprotech	120-05
Blastocidin	Life Technologies Ltd	R21001
EdU	Abcam	ab146186
Alexa Fluor™ 555 Azide, Triethylammonium Salt	ThermoFisher Scientific	A20012
RNAse A	ThermoFisher Scientific	EN0531
LDN 193189	Cambridge Bioscience	HY-12071A-10mg
PhosSTOP™ phosphatase inhibitor tablets	Roche	4906845001
DMNG	Anatrace	NG322
IPTG	Sigma-Aldrich	I6758
Bovine serum albumin	Sigma-Aldrich	A9418
Phosphate-buffered saline	Thermo Fisher Scientific	D8537-500mL
Tween20	Sigma-Aldrich	P1379-500mL
SB431542	Cell signaling	14775
1D11	BioX-Cell	BE0083
IgG1 monoclonal control antibody	BioX-Cell	BE0057

DAPI	BD Bioscience	564907
**Critical Commercial Assays**		
S-Trap micro spin columns	Protify	CO2-micro-80
Pierce™ Anti-HA Magnetic Beads	Thermo Fisher Scientific	88837
Western Lightning ECL Pro	Perkin Elmer	NEL121001EA
anti-FLAG magnetic beads	Sigma-Aldrich	M8823
anti-V5 magnetic beads	MBL	M167-11
QIAamp® DNA Blood Mini Kit	QIAGEN	51104
Monarch® Total RNA Miniprep Kit	NEB	T2010S
Luna Universal qPCR Master Mix	NEB	M3003L
Ni-NTA Agarose beads	Thermo Scientific	HisPur™ 88222
Superdex 200 Increase 10/300 GL column	GE	28-9909-44
**Deposited Data**		
Proteomics	This study; PRIDE Data Set	PXD051056<br>https://www.ebi.ac.uk/pride/archive/projects/PXD051056
Original western blot images & microscopy images	This study; Mendeley Data Set	http:// doi: 10.17632/b7r4hch9hr.1
**Experimental Models: Cell Lines & Competent cells**		
Lenti-X™ 293T Cell Line	Clontech (Takara Bio)	632180
U2OS	ECACC	92022711
HeLa	ATCC	CCL-2™
BL21-CodonPlus (DE3)-RIPL Competent cells	Agilent Technologies	230280
**Software**		
MaxQuant, version 1.6.10.43	MaxQuant	https://www.maxquant.org/
Perseus software, version 2.0.3.	MaxQuant	https://maxquant.net/perseus/
Image studio software Li-Cor v5.2	Li-Cor	https://www.licor.com/bio/image-studiolite/
FlowJo 10.8	FlowJo, LLC	https://www.flowjo.com/

GraphPad Prism 10	GraphPad	https://www.graphpad.com/scientificsoftware/prism/
ImageJ 1.53c, bundled with Java 1.8.0_172	ImageJ	https://imagej.net/software/fiji/
CellProfiler 4.2.1	CellProfiler	https://cellprofiler.org/
R 4.4.0		https://www.rproject.org/
