# Supplementary material for: Suppression of TGF-β/SMAD signaling by an inner nuclear membrane phosphatase complex
Source: Nat Commun. 2025 Apr 11;16:3474. doi: 10.1038/s41467-025-58681-x (PMC11992160; doi:10.1038/s41467-025-58681-x)
Supplement: Supplementary file 2 — Reporting Summary [file 41467_2025_58681_MOESM2_ESM.pdf]

nature portfolio

Corresponding author(s): Pedro Carvalho

Last updated by author(s): 2024/08/21

Reporting Summary

Nature Portfolio wishes to improve the reproducibility of the work that we publish. This form provides structure for consistency and transparency in reporting. For further information on Nature Portfolio policies, see our Editorial Policies and the Editorial Policy Checklist.

Statistics

For all statistical analyses, confirm that the following items are present in the figure legend, table legend, main text, or Methods section.

n/a	Confirmed	
☐	☒	The exact sample size (*n*) for each experimental group/condition, given as a discrete number and unit of measurement
☐	☒	A statement on whether measurements were taken from distinct samples or whether the same sample was measured repeatedly
☒	☐	The statistical test(s) used AND whether they are one- or two-sided<br>*Only common tests should be described solely by name; describe more complex techniques in the Methods section.*
☒	☐	A description of all covariates tested
☒	☐	A description of any assumptions or corrections, such as tests of normality and adjustment for multiple comparisons
☒	☐	A full description of the statistical parameters including central tendency (e.g. means) or other basic estimates (e.g. regression coefficient) AND variation (e.g. standard deviation) or associated estimates of uncertainty (e.g. confidence intervals)
☒	☐	For null hypothesis testing, the test statistic (e.g. *F*, *t*, *r*) with confidence intervals, effect sizes, degrees of freedom and *P* value noted<br>*Give P values as exact values whenever suitable.*
☒	☐	For Bayesian analysis, information on the choice of priors and Markov chain Monte Carlo settings
☒	☐	For hierarchical and complex designs, identification of the appropriate level for tests and full reporting of outcomes
☒	☐	Estimates of effect sizes (e.g. Cohen's *d*, Pearson's *r*), indicating how they were calculated

Our web collection on statistics for biologists contains articles on many of the points above.

Software and code

Policy information about availability of computer code

Data collection	BD FACSDiva
Data analysis	MaxQuant v1.6.3.4, v1.6.10.43; Perseus v1.5.5.3; FlowJo 10.8; Li-Cor Image Studio Lite v5.2, GraphPad Prism v10 (GraphPad)

For manuscripts utilizing custom algorithms or software that are central to the research but not yet described in published literature, software must be made available to editors and reviewers. We strongly encourage code deposition in a community repository (e.g. GitHub). See the Nature Portfolio guidelines for submitting code & software for further information.

Data

Policy information about availability of data

All manuscripts must include a data availability statement. This statement should provide the following information, where applicable:

- Accession codes, unique identifiers, or web links for publicly available datasets
- A description of any restrictions on data availability
- For clinical datasets or third party data, please ensure that the statement adheres to our policy

The Proteomics data have been deposited to the PRIDE proteomics repository with the dataset identifier PXD051056.

Research involving human participants, their data, or biological material

Policy information about studies with human participants or human data. See also policy information about sex, gender (identity/presentation), and sexual orientation and race, ethnicity and racism.

Reporting on sex and gender	N/A
Reporting on race, ethnicity, or other socially relevant groupings	N/A
Population characteristics	N/A
Recruitment	N/A
Ethics oversight	N/A

Note that full information on the approval of the study protocol must also be provided in the manuscript.

Field-specific reporting

Please select the one below that is the best fit for your research. If you are not sure, read the appropriate sections before making your selection.

☒ Life sciences ☐ Behavioural & social sciences ☐ Ecological, evolutionary & environmental sciences

For a reference copy of the document with all sections, see nature.com/documents/nr-reporting-summary-flat.pdf

Life sciences study design

All studies must disclose on these points even when the disclosure is negative.

Sample size	For immunoprecipitations in HeLa cells, the sample was determined by the amount of protein material required for label free Mass Spectrometry analysis.
Data exclusions	No data was excluded from the analyses.
Replication	All experiments, except for RT-qPCR and Edu staining, were independently repeated at least 3 times. For RT-qPCR and Edu staining experiments, biological replicates were performed.
Randomization	Randomization was not applicable and not applied in this study.
Blinding	Blinding was not applicable and not applied in this study.

Reporting for specific materials, systems and methods

We require information from authors about some types of materials, experimental systems and methods used in many studies. Here, indicate whether each material, system or method listed is relevant to your study. If you are not sure if a list item applies to your research, read the appropriate section before selecting a response.

Materials & experimental systems

n/a	Involved in the study	
☐	☒	Antibodies
☐	☒	Eukaryotic cell lines
☒	☐	Palaeontology and archaeology
☒	☐	Animals and other organisms
☒	☐	Clinical data
☒	☐	Dual use research of concern
☒	☐	Plants

Methods

n/a	Involved in the study	
☒	☐	ChIP-seq
☐	☒	Flow cytometry
☒	☐	MRI-based neuroimaging

Antibodies

Antibodies used	Rabbit Monoclonal anti-SMAD2 Cell signaling 5339S RRID:AB_10626777<br>Rabbit Monoclonal anti-pSMAD2 Cell signaling 3108S RRID:AB_490941<br>Rabbit Monoclonal anti-SMAD1 Cell signaling 6944S RRID:AB_10858882

Rabbit Monoclonal anti-pSMAD1/5/9 Cell signaling 13820S RRID:AB_2493181
Mouse Monoclonal anti-FLAG-HRP (M2) Merck Life Science UK Limited A8592 RRID:AB_439702
Rat Monoclonal anti-HA (3F10) Roche 11867423001 RRID:AB_390918
Mouse Monoclonal anti-GAPDH (1E6D9) ProteinTech 60004-1-Ig RRID:AB_2107436
Rabbit Monoclonal anti-V5 Cell signaling 13202S RRID:AB_2687461
Rabbit Monoclonal anti-SMAD4 Cell signaling 38454S RRID:AB_2728776
Rabbit Monoclonal anti-SUN1 Abcam ab124770 RRID:AB_10976056
Rabbit Polyclonal anti-SUN2 Atlas Antibodies HPA001209 RRID:AB_1080465
Rabbit Polyclonal anti-MAN1 Atlas Antibodies HPA076986 RRID:AB_2686821
Rabbit Monoclonal anti-p21 Cell signaling 2947S RRID:AB_823586
Rabbit Monoclonal anti-Lipin1 Cell signaling 5195S RRID:AB_10694491
Peroxidase AffiniPure Goat Anti-Mouse IgG, light chain specific Jackson ImmunoResearch 115-035-174, RRID:AB_2338512
Peroxidase IgG Fraction Monoclonal Mouse Anti-Rabbit IgG, light chain specific Jackson ImmunoResearch 211-032-171 RRID:AB_2339149
Peroxidase AffiniPure Goat Anti-Rat IgG, light chain specific Jackson ImmunoResearch 112-035-175 RRID:AB_2338140
Self-raised anti-CTDNEP1 antibody Eurogentec

Validation

Primary antibodies were validated by including negative controls, such as isotype or KO samples, and/or by including positive controls, such as overexpression samples. The correct size of the bands on western blots were checked using markers. Antibody validation information from the manufacturer's websites was also consulted.

Eukaryotic cell lines

Policy information about cell lines and Sex and Gender in Research

Cell line source(s)

HeLa cells were obtained from the ATCC. U2OS cells were obtained from the ECACC. The Lenti-X 293T cell line for production of lentivirus was obtained from TakaraBio

Authentication

Authenticity was guaranteed by the supplier.

Mycoplasma contamination

Cells were routinely checked in-house for mycoplasma using Lonza MycoAlert mycoplasma detection kit

Commonly misidentified lines
(See ICLAC register)

N/A

Plants

Seed stocks

N/A

Novel plant genotypes

N/A

Authentication

N/A

Flow Cytometry

Plots

Confirm that:

☒ The axis labels state the marker and fluorochrome used (e.g. CD4-FITC).

☒ The axis scales are clearly visible. Include numbers along axes only for bottom left plot of group (a 'group' is an analysis of identical markers).

☒ All plots are contour plots with outliers or pseudocolor plots.

☒ A numerical value for number of cells or percentage (with statistics) is provided.

Methodology

Sample preparation

For EdU incorporation assay to assess cell proliferation, cells were seeded in 6-well plates for 24h prior to 30 minutes incubation with 10µM EdU (Abcam, #ab146186). Cells were then harvested, washed with PBS, and fixed with 70% Ethanol overnight. Fixed cells were pelleted by centrifuging at 1000xg for 5min, washed with PBS, and permeabilized with PBSTri-BSA (PBS, 0.1% Triton X-100, 1% BSA) on ice for 15min. Permeabilized cells were pelleted and washed twice with PBST-BSA (PBS, 0.1% Tween20, 1% BSA). EdU present in the cells were stained by Click-IT reaction (2mM $CuSO_4$, 10mM Sodium Ascorbate, 10µM Alexa Fluor™ 555 Azide, Triethylammonium Salt (ThermoFisher Scientific #A20012) in PBS) at room temperature for 1h in dark. After Click-IT reaction, cells were pelleted and washed twice with PBST-BSA. DAPI staining was then performed in

DAPI containing solution (PBS, 0.1% BSA, 1mg/mL RNAse A (ThermoFisher Scientific, # EN0531), 1µg/mL DAPI (BD Bioscience, #564907)) for 1h in room temperature in dark. Next, cells were analyzed using a BD LSRFortessa X-20 flow cytometer. For each condition, 30,000 cells were measured and FACS data was analyzed using FlowJo v10.

Instrument	BD LSRFortessa™ X-20
Software	BD FACSDiva for collection. FlowJo v10 for analysis
Cell population abundance	N/A
Gating strategy	At least 10000 cells per sample were measured gated on the main population in the FSC/SSC plot.

☐ Tick this box to confirm that a figure exemplifying the gating strategy is provided in the Supplementary Information.
